# Expansion and Characterization of Human Melanoma Tumor-Infiltrating Lymphocytes (TILs)

**DOI:** 10.1371/journal.pone.0013940

**Published:** 2010-11-10

**Authors:** Linh T. Nguyen, Pei Hua Yen, Jessica Nie, Nicole Liadis, Danny Ghazarian, Ayman Al-Habeeb, Alexandra Easson, Wey Leong, Joan Lipa, David McCready, Michael Reedijk, David Hogg, Anthony M. Joshua, Ian Quirt, Hans Messner, Patricia Shaw, Michael Crump, Eran Sharon, Pamela S. Ohashi

**Affiliations:** 1 Campbell Family Institute for Breast Cancer Research, Ontario Cancer Institute, University Health Network, Toronto, Canada; 2 Department of Pathology, Princess Margaret Hospital, University Health Network, Toronto, Canada; 3 Department of Surgical Oncology, Princess Margaret Hospital, University Health Network, Toronto, Canada; 4 Department of Medical Oncology/Hematology, Princess Margaret Hospital, University Health Network, Toronto, Canada; 5 Departments of Medical Biophysics and Immunology, University of Toronto, Toronto, Canada; New York University, United States of America

## Abstract

**Background:**

Various immunotherapeutic strategies for cancer are aimed at augmenting the T cell response against tumor cells. Adoptive cell therapy (ACT), where T cells are manipulated ex vivo and subsequently re-infused in an autologous manner, has been performed using T cells from various sources. Some of the highest clinical response rates for metastatic melanoma have been reported in trials using tumor-infiltrating lymphocytes (TILs). These protocols still have room for improvement and furthermore are currently only performed at a limited number of institutions. The goal of this work was to develop TILs as a therapeutic product at our institution.

**Principal Findings:**

TILs from 40 melanoma tissue specimens were expanded and characterized. Under optimized culture conditions, 72% of specimens yielded rapidly proliferating TILs as defined as at least one culture reaching ≥3×10^7^ TILs within 4 weeks. Flow cytometric analyses showed that cultures were predominantly CD3+ T cells, with highly variable CD4+:CD8+ T cell ratios. In total, 148 independent bulk TIL cultures were assayed for tumor reactivity. Thirty-four percent (50/148) exhibited tumor reactivity based on IFN-γ production and/or cytotoxic activity. Thirteen percent (19/148) showed specific cytotoxic activity but not IFN-γ production and only 1% (2/148) showed specific IFN-γ production but not cytotoxic activity. Further expansion of TILs using a 14-day “rapid expansion protocol” (REP) is required to induce a 500- to 2000-fold expansion of TILs in order to generate sufficient numbers of cells for current ACT protocols. Thirty-eight consecutive test REPs were performed with an average 1865-fold expansion (+/− 1034-fold) after 14 days.

**Conclusions:**

TILs generally expanded efficiently and tumor reactivity could be detected in vitro. These preclinical data from melanoma TILs lay the groundwork for clinical trials of ACT.

## Introduction

Recent experimental evidence solidifies the concept that the immune system surveys the body for tumors and can eliminate them [1], [2]. Many studies have identified the presence of tumor-specific T cells in peripheral blood, tumor-draining lymph nodes and within tumors of cancer patients [3]–[5]. However, it is clear that the natural anti-tumor T cell response is not always sufficient to prevent tumor progression. Various immunotherapeutic approaches for cancer have been developed, with the aim of enhancing the anti-tumor T cell response. Some approaches focus on amplifying endogenous responses, and to this end, various vaccination strategies have been explored [6]. Indeed, some peptide vaccines have succeeded in expanding tumor-reactive T cells in patients when combined with immunological adjuvants [7]. Recently, a peptide vaccine showed potential in improving progression-free survival in a randomized clinical trial [8]. Other strategies are aimed at disrupting negative regulators of the T cell response, such as blockade of the cytotoxic T lymphocyte antigen-4 (CTLA-4) molecule, which is currently in late-phase clinical trials [9]–[11], or the more recent development of blocking antibodies against the programmed death-1 (PD-1) molecule [12]–[14].

Another approach, adoptive T cell therapy, focuses on amplifying patients' T cells ex vivo followed by autologous re-infusion. Different sources of T cells have been evaluated in clinical trials for adoptive cell therapy including, for melanoma: T cell clones [15]–[18], T cells expanded from tumor-infiltrating lymphocytes (TILs) [19]–[32], or peripheral blood T cells retrovirally transduced with T cell receptors (TCRs) that recognize tumor-associated antigens [33]. Clinical trials based on adoptive transfer of TILs have been performed for other types of cancer as well (for example, [34]–[37]). Collectively these trials demonstrate that adoptive transfer of TILs is associated with minimal toxicities. Furthermore, some of these studies provide evidence that TILs are clinically active.

Melanoma is one of the more frequently studied cancers in the field of immunotherapy, for reasons including accessibility of lesions, the discovery of melanoma-associated antigens and detection of tumor-specific T cells. In addition, metastatic melanoma has a poor prognosis with a five-year survival of less than 2% and very limited treatment options. Approved treatments for metastatic melanoma include interleukin-2 (IL-2) and chemotherapy. High-dose IL-2 therapy has (at best) an overall response rate of 16% and a complete response rate of 6% [38]. Dacabarzine-based chemotherapy has an overall response rate of 7.5%, with very few complete responders and almost no long-term survivors [39].

Recently, high clinical response rates have been observed by the Rosenberg group, where metastatic melanoma patients were treated with TIL-based protocols in a series of trials. In these protocols, patients were given non-myeloablative lymphodepleting chemotherapy (cyclophosphamide and fludarabine) immediately prior to infusion of TIL (10^10^–10^11^ cells) and high-dose IL-2 therapy. Using this protocol, the objective clinical response rate by RECIST criteria was a notable 49% (21/43 patients) [29]–[31]. When myeloablative total body irradiation (2 or 12 Gray) was added to the treatment protocol, a trend of higher clinical response rates with increasing intensity of lymphodepletion was observed (52% (13/25) objective response rate with 2 Gy and 72% (18/25) objective response rate with 12 Gy) [31]. Other groups have demonstrated that lymphodepletion prior to T cell transfer can improve T cell persistence compared with T cell transfer without prior lymphodepletion [40]. Modified methods of TIL preparation have also been explored in an effort to improve responses as well as to streamline the cell production process [41], [42].

Adoptive cell therapy using TILs is associated with some of the highest clinical response rates for metastatic melanoma to date. Clearly, combination therapy with other agents or perhaps modification of TILs has the potential to further improve clinical responses, decrease the number of TILs needed for therapy and/or decrease the toxicities associated with cyclophosphamide/fludarabine preparative regimens. Therefore we were interested in developing clinical trials using TIL-based adoptive cell therapy for melanoma. As a first step, we performed preclinical work to expand and characterize melanoma TILs. Using procedures developed by the Rosenberg group, we have analyzed TILs from 40 melanoma specimens and established standard operating procedures for generating TILs for therapeutic use.

## Results and Discussion

### Tissue specimens

A total of 40 melanoma tissue specimens were obtained from patients undergoing surgical procedures under standard-of-care. Patient characteristics are listed in Table 1. Three patients each underwent surgical procedures on two occasions from which we obtained tissue: Specimen #M2 and M7 were from the same patient; specimen #M30 and M33 were from the same patient; specimen #M38 and M41 were from the same patient. Most specimens were obtained from subcutaneous lesions (n = 21), many from nodal metastases (n = 13), and a few from other sites (lung, liver, abdominal wall, pelvic cavity) (n = 6). The predominant melanoma subtype was superficial spreading melanoma (n = 30) (data not shown), which reflects the predominance of this melanoma type in the general North American population [43]. The remaining histological types were nodular, acral lentiginous or lentigo maligna melanoma (n = 7) or undetermined (n = 3) (data not shown). Twenty specimens were from male patients, and twenty from female patients. The average age at the time of tissue acquisition was 60 years (+/− 17 years; median 61 years). Patients were heavily skewed towards late stage disease, and thus matched the target demographic for potential future adoptive cell therapy clinical trials. There were 18 patients with stage IV melanoma at the time of tissue acquisition, 18 with stage III, 4 with stage II and none with stage I disease. Typing was performed at the HLA-A locus in order to determine the appropriate panel of target cells for assays of TIL function. Sixteen specimens were HLA-A*0201, which is the most common HLA-A type amongst Caucasians [44].

**Table 1:** Patient characteristics.

Specimen #	Gender	Age	Site of specimen^1^	Stage^2^	Treatment(s) prior to surgery^3^	HLA-A type	
M1	F	55	LN	IV	chemo	0201/03	
M2	F	62	SC	IV	IFN, chemo, IL-2	0201/01	
M3	M	49	SC	IV	IFN, chemo	0201/01	
M4	F	55	LN	IV	-	32/32	
M5	M	40	LN	IV	IFN, IL-2	33/68	
M7	F	62	SC	IV	IFN, chemo	0201/01	
M8	M	61	SC	III	-	2402/68	
M9	F	29	LN	IV	IFN	0101/32	
M10	F	51	Lung	IV	-	0201/03	
M13	M	34	LN	III	-	2402/68	
M15	M	42	SC	III	-	0101/0101	
M16	M	67	SC	III	-	0205/11	
M17	M	39	SC	IV	IFN	0101/26	
M18	M	58	SC	IV	IFN, chemo	nd	
M19	M	46	SC	IV	-	nd	
M20	F	88	SC	III	-	0201/32	
M21	M	62	ABD	IV	-	2402/26	
M22	F	82	SC	II	-	30/68	
M23	M	69	LN	IV	-	0101/11	
M24	M	76	SC	II	-	0101/0201	
M25	M	78	SC	III	-	0101/0201	
M26	F	33	SC	III	-	0206/0206	
M27	F	28	LN	III	IFN	0207/11	
M28	F	72	SC	II	-	0301/26	
M29	F	77	SC	III	-	0201/11	
M30	M	82	LN	III	-	0201/0202	
M31	M	66	SC	III	IFN	0201/24	
M32	M	63	LN	III	-	0301/32	
M33	M	83	LN	III	-	0201/0202	
M34	F	83	SC	III	-	0101/0205	
M35	F	51	LN	III	-	0101/0301	
M36	M	61	Liver	IV	IFN	11/29	
M37	F	49	LN	III	IFN	0201/2402	
M38	F	39	PELV	IV	-	0201/0201	
M39	M	79	SC	III	-	0216/33	
M40	F	95	SC	III	-	0201/3002	
M41	F	39	Lung	IV	-	0201/0201	
M43	F	61	LN	IV	IFN, chemo, IL-2	0301/68	
M44	F	58	Lung	IV	-	11/11	
M45	M	61	SC	II	-	0101/0301	

1 The anatomical site from which the melanoma tissue specimen was obtained is indicated. LN, lymph node. SC, subcutaneous. ABD, abdominal wall. PELV, pelvic cavity.

2 The disease stage at the time of surgery is indicated.

3 Any non-surgical treatment for melanoma that occurred anytime prior to tissue acquisition is indicated. IFN, interferon-α therapy. IL-2, high-dose interleukin-2 therapy.

nd, not done.

### TIL culturing

Tissues were processed by a variety of methods in preparation for culturing TILs, depending in part on the amount of tissue available. The specimen sizes obtained for this study varied widely, from core biopsies to 3 cm^3^-sized samples. Single cell suspensions were obtained by 1) enzymatic dissociation, 2) fine needle aspirates, 3) mechanical dissociation using a Medimachine. Cells obtained by these methods were plated and TILs were expanded as described in the Materials and Methods. In addition, small tissue fragments were also plated for TIL growth. In general, we aimed to initiate TIL cultures from at least 8 tissue fragments and 10×10^6^ cells from enzymatically dissociated tissue; however this varied depending on the size of the specimen.

Generally, TILs emerged from tissue fragments or began to proliferate from single cell suspensions within 1–2 weeks of culture initiation, and other cells such as tumor cells disappeared from the cultures during that same time period. We observed that expansion of TILs was more efficient following enzymatic dissociation or plating tissue fragments (data not shown) and therefore these methods will be used for processing tissues for our planned clinical trials. Multiple independent bulk cultures of TILs were maintained for each tissue specimen, with each independent culture originating from approximately 1–2 parental wells. Each culture was generally expanded for 4 weeks or until the minimum number of TILs that would be needed for therapeutic protocols were obtained (minimum 3×10^7^ TILs).

**Figure 1:**
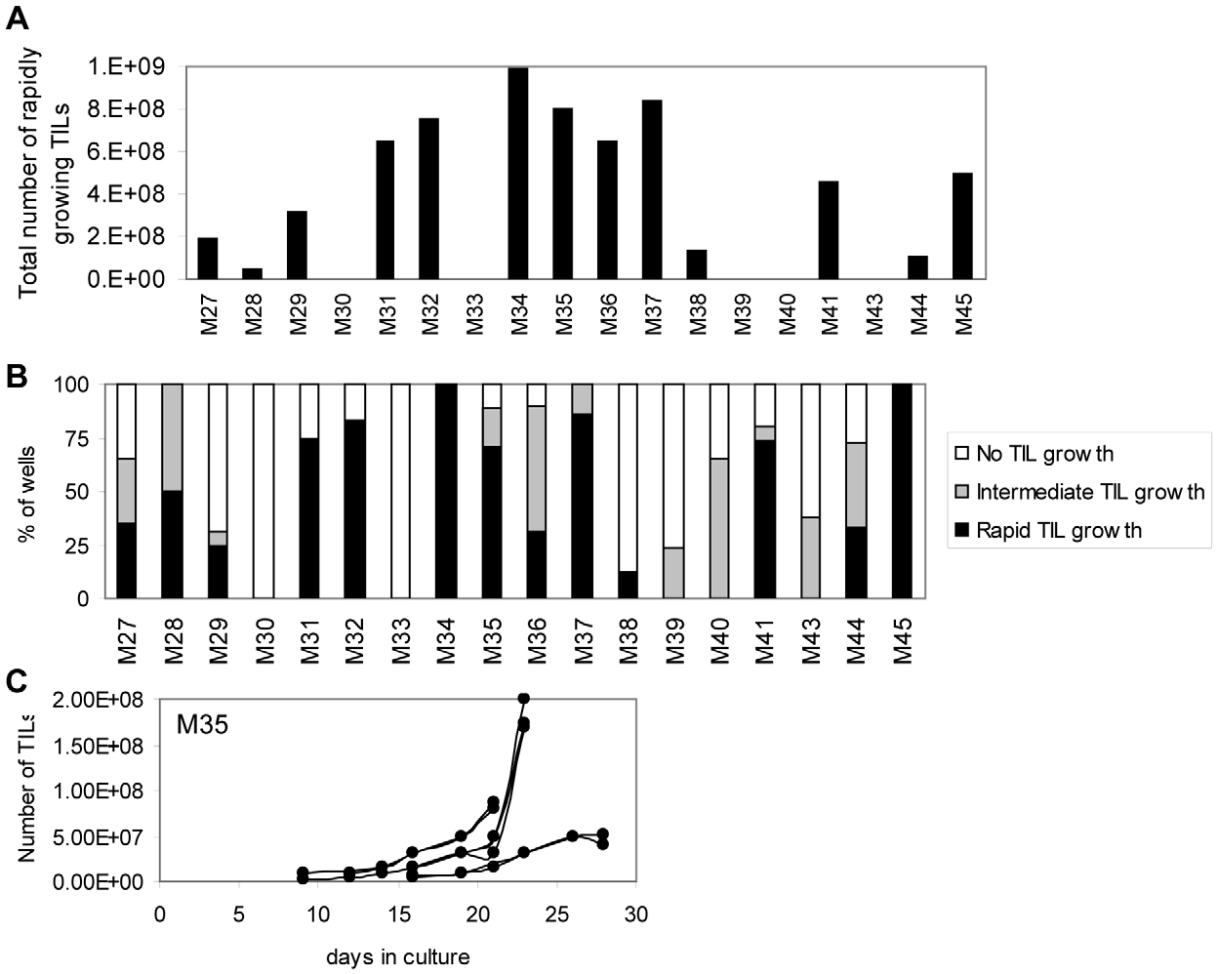
Growth of TILs in vitro. Single cell suspensions or tissue fragments from melanoma specimens were cultured in medium containing 10% “in-house” human plasma and 6000 IU/ml IL-2. (A) For each tissue specimen (x-axis), the total number of TILs from all cultures that exhibited “rapid growth” (≥30×10^6^ TILs within 4 weeks) were enumerated. (B) The proportion of wells (plated on day 0) that exhibited rapid TIL growth (≥30×10^6^ TILs within 4 weeks), intermediate TIL growth (<30×10^6^ TILs within 4 weeks) and no TIL growth are shown for each tissue specimen. (C) Growth curves for seven TIL cultures from a representative tissue specimen (M35) are shown. Each line represents an independent TIL culture.

Our initial cultures were maintained in complete medium containing commercially available human serum. We found that TILs could be expanded from most melanoma tissues, but growth rates were overall quite slow, with only 7 of 22 specimens (32%) yielding at least one culture with ≥3×10^7^ cells within 4 weeks. We therefore began to use plasma from healthy donors that was obtained and processed at our own institution, instead of commercially available serum. Growth rates of TIL cultures greatly improved, with 13 of 18 tissue specimens (72%) yielding at least one TIL culture reaching ≥3×10^7^ TILs within 4 weeks. In fact, most specimens yielded multiple cultures exhibiting rapid growth rates and therefore a total number of cells far exceeding the 3×10^7^ minimum (Figure 1A). We did not observe any differences in TIL expansion between specimens obtained from different anatomical sites (i.e. subcutaneous, visceral or nodal lesions) (data not shown). For each of the 18 tissue specimens, the percentage of parental wells that yielded TIL cultures with rapid growth rates (defined as reaching ≥3×10^7^ TILs within 4 weeks), intermediate growth rates (<3×10^7^ TILs within 4 weeks), or no TIL growth, were enumerated for each tissue specimen (Figure 1B). Interestingly, independent wells derived from the same tissue specimen often exhibited differential expansion of TILs. An example of the growth rates of representative “rapidly growing” independent TIL cultures derived from tissue specimen #M35 is shown in Figure 1C. The variability in growth rates may be due to different cellular composition in different tissue fragments, or stochastic differences between cells plated in different parental wells.

**Figure 2:**
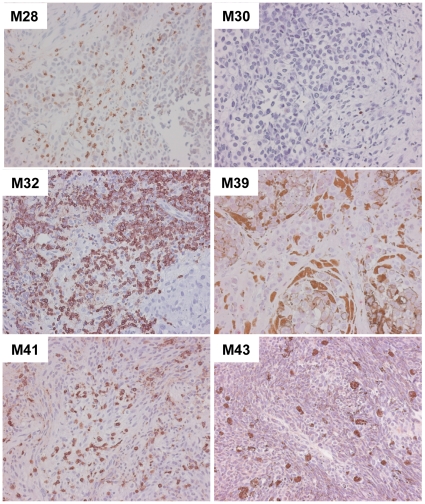
Immunohistochemistry for CD3. A piece of each tissue specimen from which TIL culturing was attempted was stained for CD3. Left column: dense TIL infiltration in representative tissue specimens that yielded robust TIL growth in vitro (M28, M32, M41). Right column: paucity of TILs in tissue specimens that yielded no or poor TIL growth in vitro (M30, M39, M43). Note that the dark staining in M39 is from pigmented melanoma cells and melanophages and the dark staining in M43 is from melanophages. Original magnification: 20x.

Immunohistochemistry (IHC) was performed on a piece of each tissue specimen. Most samples exhibited infiltration by CD3+ T cells. Notably, amongst specimens that were cultured using “in-house” plasma, 4 of the 5 specimens that exhibited the poorest TIL growth in vitro (M30, 33, 39, 43; see Figure 1A, B) also showed a paucity of CD3+ T cells by IHC compared with specimens that exhibited efficient TIL growth (Figure 2). The fifth specimen with poor TIL growth (M40) proved to be highly necrotic upon microscopic examination (data not shown). Although our methods did not permit direct comparisons of TIL growth rates between different specimens, this qualitative observation indicates that if T cells are present in situ, then they generally have the ability to expand ex vivo in the presence of IL-2.

### Phenotype of cultured TILs

**Figure 3:**
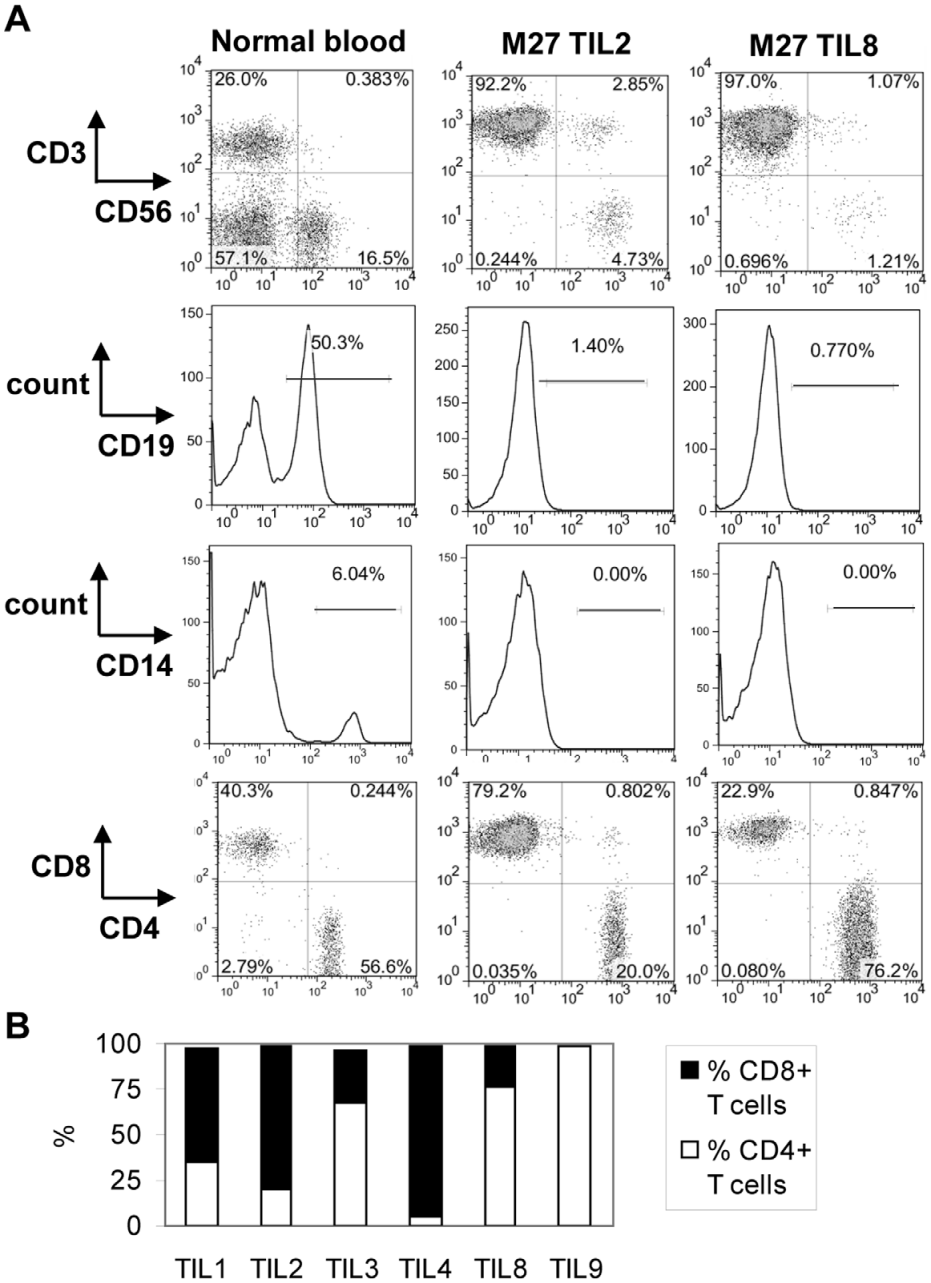
Flow cytometric analysis of TILs. (A) Profiles of representative TIL cultures (specimen M27, TIL cultures 2 and 8) stained for CD3, CD56, CD19, CD14, CD4 and CD8 are shown. Profiles from peripheral blood cells (PBCs) from a healthy donor are shown for comparison (“normal blood”). PBC profiles are gated on either lymphocytes (CD56xCD3, CD19, CD4xCD8) or total PBCs (CD14). TIL profiles are gated on live TILs based on forward scatter and side scatter for all plots. All CD4xCD8 profiles are also gated on CD3+ cells. (B) The proportion of CD4+ and CD8+ T cells (gated on CD3+ cells) are shown for all six independent TIL cultures derived from tissue specimen M27.

Healthy TIL cultures consisted of cells with morphological features typical of activated T cells, with some clusters of rapidly proliferating TILs (data not shown). The presence or absence of various immune cell types was evaluated by flow cytometric analysis for markers including CD19 (B cells), CD14 (monocytes) and CD3 (T cells). An example of these profiles is shown in Figure 3A. As expected, CD3+ T cells were the predominant cell type. CD19+ and CD14+ cells were consistently absent. A minority of TIL cultures showed expression of the prototypic NK cell marker CD56 on a subset of cells. TIL cultures contained a median of 4.16% CD56+ CD3− cells (mean 10% +/− 13%). CD56 was also co-expressed with CD3 on occasion (median 5.6% of TILs, mean 10% +/− 11%), however when selected cultures were stained for the invariant TCR chains expressed by human NKT cells (V_α_24 and Vβ11), these chains were absent (data not shown). Therefore co-expression of CD56 and CD3 on a subset of cells likely reflects the previously reported expression of CD56 as a T cell activation marker [45] and not the presence of NKT cells. Flow cytometric analysis also revealed that TIL cultures exhibited highly heterogeneous ratios of CD4+:CD8+ T cells. An analysis of CD3+ cells from all healthy TIL cultures from patients #M27 - M45 (cultured in medium containing “in-house” human plasma) revealed an average of 37% +/− 28% CD4+ T cells (median 33%) and 52% +/− 30% CD8+ T cells (median 52%). Even independent TIL cultures established from the same tissue specimen often showed variable CD4+:CD8+ T cell ratios (Figure 3B). HLA-DR was also examined as a T cell activation marker and was found to be upregulated in all TIL cultures, as would be expected for proliferating T cells (data not shown).

### Function of cultured TILs

Although lymphocytes found within non-lymphoid tissues, including tumors, are often enriched for T cells that are able to recognize specific tissue-associated antigens, we sought to determine whether “tumor reactivity” of cultured TILs could be detected in vitro. The ability of TILs to recognize and respond to tumors was assessed using two assays: 1) release of the proinflammatory cytokine IFN-γ by TILs upon encounter with tumor cells or tumor-associated antigens and 2) cytolytic activity of TILs against tumor cells or tumor-associated antigens. Whenever available, TILs were assayed against autologous tumor cells (cryopreserved directly from tumor tissue or cultured from tumor tissue). “Surrogate” target cells were also used when appropriate. One set of surrogate target cells consisted of a panel of established melanoma cell lines: 526 mel, 624 mel, 888 mel, 938 mel. Because the recognition of target cells is restricted by having matched HLA loci between the target cell and the T cell, these melanoma cell lines were either used as “test” target cells (in the case of an HLA-A locus match between a given melanoma cell line and patient TILs) or negative control target cells (in the case of an HLA-A locus mismatch between a given melanoma cell line and patient TILs). For TILs from HLA-A*0201+ patients, T2 cells were also used as targets. The T2 cell line is an HLA-A*0201+ lymphoblastoid cell line that is deficient for the “transporter associated with antigen processing” protein (TAP). This deficiency allows for efficient “loading” of these cells with HLA-A*0201-restricted peptides. For these assays, T2 cells were loaded with peptides from one of two melanoma-associated antigens: Melan-A/MART-1-derived peptide (aa27–35) or gp100-derived peptide (aa209–217). Both of these differentiation antigens are expressed on >90% of melanomas [46].

An example of results for IFN-γ secretion are shown in Table 2, where eight independent bulk TIL cultures (named TIL1 – TIL8) from tissue specimen #M31 (HLA-A*0201+) were each co-cultured with a panel of target cells. TILs were also stimulated with PMA and ionomycin as a positive control for non-specific IFN-γ secretion. Previously characterized HLA-A*0201-restricted control T cell lines showed the expected reactivity against Melan-A-pulsed T2 cells and gp100-pulsed T2 cells. The results showed that all eight TIL cultures secreted IFN-γ in response to autologous tumor cells. In addition, Melan-A-specific IFN-γ production was detected from TIL cultures #1 (TIL1), TIL5, TIL7 and TIL8. Furthermore, TIL8 also produced IFN-γ in response to gp100 peptide-pulsed targets. Background IFN-γ levels were below detection in cultures containing TIL alone, or TIL co-cultured with T2 cells not loaded with peptide.

**Table 2:** IFN-γ production after co-culture of TILs with a panel of stimulator cells.

	no stimulators	PMA + ionomycin	526 mel	888 mel	T2	T2 + Melan-A	T2 + gp100	Autologous tumor cells
Melan-A-specific control	<	11075	<	<	<	1525	<	2025
gp100-specific control	<	16750	10050	<	<	<	13200	20800
TIL1	<	15900	<	<	<	4350	<	4800
TIL2	<	16225	<	<	<	<	<	3400
TIL3	<	17925	<	<	<	<	<	9225
TIL4	<	16050	<	<	<	<	<	2050
TIL5	<	19400	725	<	<	3450	<	10575
TIL6	<	16200	<	<	<	<	<	5075
TIL7	<	15525	<	<	<	800	<	2875
TIL8	<	15775	<	<	<	1150	3650	2325

Eight independent TIL cultures (TIL1-8) from specimen #M31 were co-cultured with various stimulator cells. Supernatants were assayed for IFN-γ production by ELISA. Concentrations are shown in pg/ml.

“<” indicates IFN-γ below the detection limit of 195 pg/ml.

**Figure 4:**
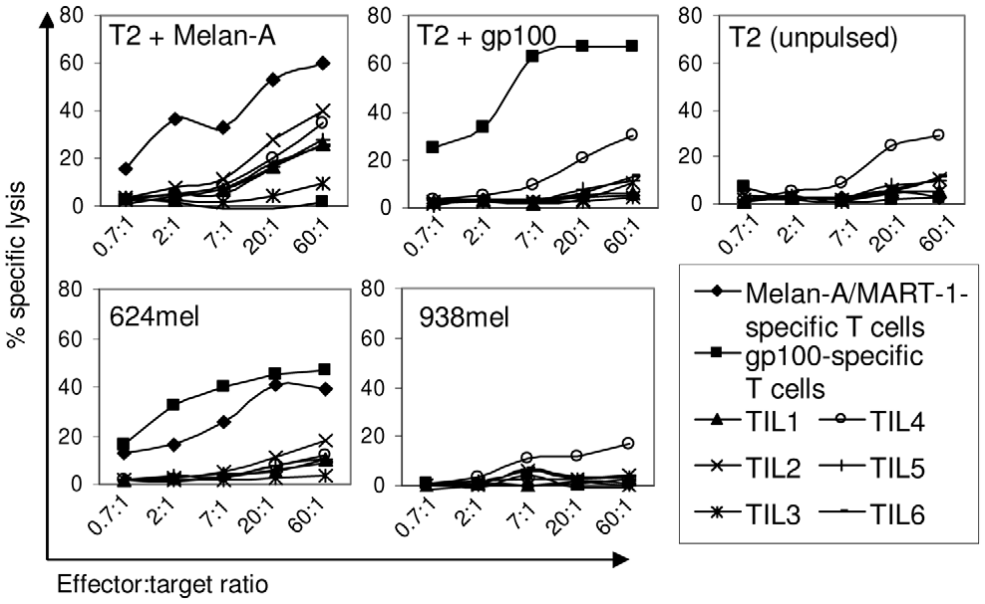
Cytotoxicity assay. Six TIL cultures derived from specimen M25 were assayed for cytotoxic activity against the indicated ^51^chromium-loaded target cells. Melan-A/MART-1- and gp100-specific T cell lines were used as positive controls.

An example of a cytotoxic T lymphocyte (CTL) assay is shown in Figure 4. Six independent bulk TIL cultures (named TIL1-TIL6) from tissue specimen #M25 (HLA-A*0201+) were each assayed for their ability to lyse a panel of ^51^chromium-loaded target cells. Previously characterized HLA-A*0201-restricted control T cell lines showed the expected reactivity against Melan-A-pulsed T2 cells and gp100-pulsed T2 cells. For this specimen, autologous tumor cells were not available. However, four of six cultures exhibited cytolytic activity against T2 cells loaded with Melan-A peptide (defined as ≥20% specific lytic activity at a 60∶1 effector:target cell ratio). An additional TIL culture (TIL4) also lysed Melan-A-loaded T2 cells, however this TIL culture also lysed T2 cells not loaded with peptide and therefore was considered non-specific. None of the TIL cultures lysed the HLA-A-unmatched melanoma cell line 938 mel.

**Figure 5:**
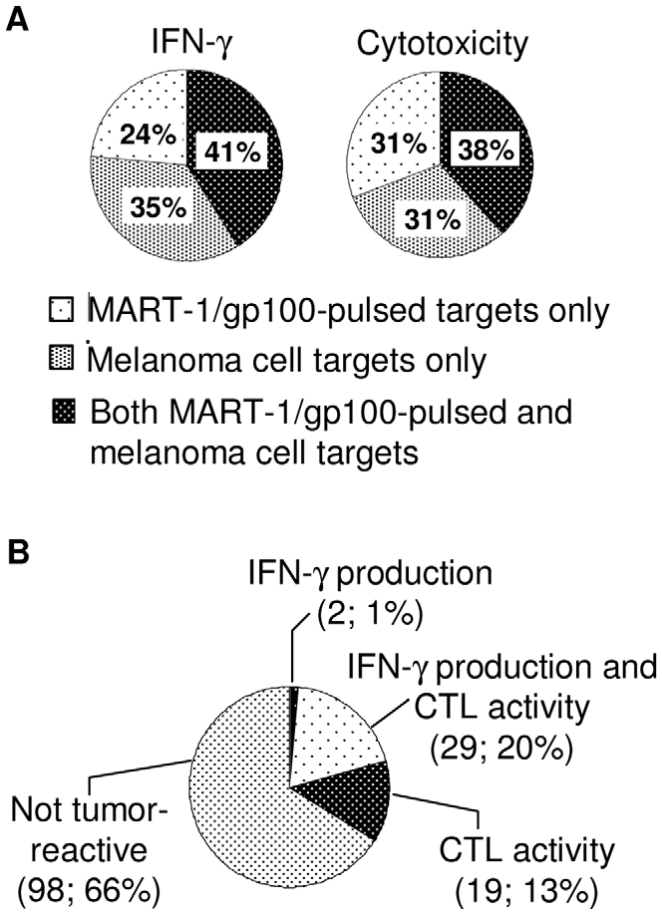
Tumor reactivity of TILs. (A) Summary of data from all HLA-A*0201+ TIL cultures that exhibited reactivity against melanoma cells (i.e. autologous tumor cells or HLA-A*0201+ melanoma cell lines) and/or peptide-pulsed T2 cells (i.e. MART-1 or gp100 peptides). The proportion of TIL cultures showing reactivity against MART-1/gp100-pulsed T2 cells only, melanoma cells only, or both MART-1/gp100-pulsed and melanoma cells is shown. IFN-γ data are from 17 TIL cultures; CTL data are from 26 TIL cultures. (B) Results from functional assays of all TIL cultures (for which relevant targets were available) are summarized. The number and proportion of TIL cultures exhibiting tumor reactivity (based on IFN-γ production, CTL activity, or both) or not exhibiting tumor reactivity are shown.

Interestingly, the IFN-γ and cytotoxicity data from some of the HLA-A*0201+ TIL cultures show discordance in their reactivity against peptide-pulsed target cells and melanoma cells (Figure 5A). Some cultures exhibited reactivity against peptide-pulsed T2 cells (Melan-A or gp100) but not against autologous melanoma or HLA-A*0201+ melanoma cell lines. This may reflect a lower level of endogenous Melan-A or gp100 peptides presented on the surface of melanoma cells compared with levels of these peptides exogenously loaded onto T2 cells. This hypothesis is supported by peptide titration experiments, where pulsing 624 mel cells with 1 µM or 10 µM of Melan-A peptide led to increased IFN-γ production by TILs, and conversely, decreasing the concentration of Melan-A peptide on T2 cells led to decreased IFN-γ production (data not shown). Other cultures showed reactivity against melanoma cells but not peptide-pulsed T2 cells. These TILs may recognize other peptide epitopes of the Melan-A or gp100 proteins; it is also likely that the antigen specificity of these TILs is much broader and may encompass other antigens.

All TIL cultures that yielded sufficient numbers of cells and for which appropriate target cells were available were assayed for tumor reactivity. Of the 40 tissue specimens that were obtained, 33 specimens yielded sufficient TILs to perform both the IFN-γ and CTL assays described above. Of these, appropriate target cells were available for assaying TILs from 31 specimens. A positive signal from either IFN-γ production or CTL activity was taken as an indication of “tumor reactivity”. From these assays, 15 specimens yielded at least one TIL culture that exhibited tumor reactivity in vitro. This result is comparable with the experience of two other groups using similar methods [32], [41].

For each of the specimens from which TIL cultures could be established, between one and eight independent bulk cultures were obtained, for a total of 241 TIL cultures from all specimens. Appropriate target cells were available to assay for both IFN-γ production and CTL activity for 148 of these TIL cultures. Results from these analyses are summarized in Figure 5B. Overall, 34% of the TIL cultures assayed showed evidence of tumor reactivity by IFN-γ production, CTL activity, or both readouts. This proportion is similar to the proportion of tumor reactive TILs reported by other groups [41]. The majority of the tumor reactive TIL cultures were identified based on both read-outs (20% with IFN-γ production and CTL activity). Interestingly, TIL cultures exhibiting tumor reactivity based on only one of the two read-outs were more likely to exhibit specific CTL activity and not IFN-γ production, and not the opposite. The use of CTL activity as a read-out enabled the identification of 19 additional tumor reactive TIL cultures that were not identified based on IFN-γ production. The mechanism for this differential effector function is not clear. However, a similar observation was previously reported where low avidity TCR interactions in the absence of co-stimulation could trigger target cell lysis but not the production of cytokines (in that case, IL-2) [47]. Since cytokine production requires de novo gene transcription whereas cytotoxic activity utilizes pre-formed lytic granules, one could speculate that a low TCR signal strength is sufficient for cytotoxicity but not cytokine production. Furthermore, signaling pathways downstream of TCR triggering in some TILs may be muted, in a similar manner as described by Ohlen et al [48]. We also noted that functional assays of TILs that had been cultured in medium containing “in-house”-generated human plasma yielded a similar proportion of tumor-reactive cultures compared with TILs that had been cultured in medium containing commercially available human serum (data not shown). This indicates that the source of the blood product used to expand TIL does not affect TIL reactivity.

### Rapid expansion protocol (REP)

**Figure 6:**
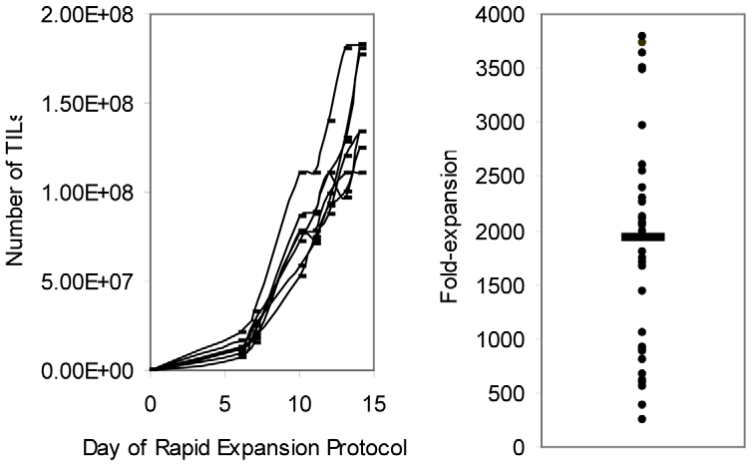
Rapid expansion protocol (REP). (A) The expansion rate of the six TIL cultures derived from specimen M32 is shown. REPs were initiated with 2×10^5^ TILs on day 0 (with 4×10^7^ irradiated allogeneic PBMCs, 30 ng/ml OKT3 and 3000 IU/ml IL-2) and maintained as described in the Materials and Methods. Each line represents an individual TIL culture. (B) The fold-expansion over 14 days is shown for 38 consecutive REPs. Each symbol shows the fold-expansion for one REP. The solid line shows the mean fold-expansion.

Currently, various protocols for adoptive T cell therapy all involve the infusion of large numbers of cells (>10^10^). One efficient method for rapidly expanding TILs for therapeutic use is referred to as the “rapid expansion protocol” (REP) [49]. In the REP, TILs are cultured with soluble anti-CD3 monoclonal antibody (OKT3 clone), human recombinant IL-2 and feeder cells. The feeder cells are peripheral blood mononuclear cells from healthy donors that are irradiated prior to culture in order to prevent their proliferation during the REP. This method has been shown to induce 500- to 2000-fold expansion of TILs after 14 days. In order to establish this method in our laboratory, selected TIL cultures that had undergone the initial expansion phase with IL-2 for several weeks were subjected to the REP. Figure 6A shows representative REP growth curves for seven independent TIL cultures from tissue specimen #M32. For this particular expansion, 2×10^5^ cells from each TIL culture (obtained after culture in IL-2 for several weeks following tissue processing) were seeded in the REP. Under optimized conditions, we have performed 38 REPs for various TIL cultures, with an average fold-expansion of 1865 +/− 1034 (Figure 6B). Therefore, the TILs that we have generated have the capacity to expand to doses required for therapeutic protocols.

### Conclusion

This study included preclinical work in preparation for clinical trials of adoptive cell therapy using melanoma TILs. Conditions for the expansion and characterization of TILs have been optimized at our institution and the data show that the parameters associated with the TIL cultures (growth rate, phenotype and function) are comparable with other groups. The data also show that the use of cytotoxic activity as a read-out, in addition to IFN-γ production, may improve the identification of tumor reactive TILs. This study supports the translation of this adoptive cell therapy approach to multiple centers. The design of clinical protocols using TILs at our institution is underway.

## Materials and Methods

### Ethics Statement

This study was conducted according to the principles expressed in the Declaration of Helsinki. The study was approved by the Institutional Review Board of the University Health Network and Mount Sinai Hospital (protocol numbers 05-0495-T, 07-0074-E, 05-0956-C, 06-0129-CE). All patients provided written informed consent for the collection of samples and subsequent analysis.

### Tissue specimens

Tissues were obtained from melanoma patients undergoing standard-of-care surgical procedures. All patients gave written consent under approved institutional research ethics board (REB) protocols (University Health Network (UHN) REB 05-0495-T, Mount Sinai Hospital REB 07-0074-E). There were no restrictions (e.g. stage, prior treatment, etc) on tissues included for this study other than confirmation of melanoma by Pathology review of H&E slides from the specimen taken for research. Tissue was obtained fresh and immediately transported to the laboratory in sterile saline for processing.

### Blood products

Human serum, plasma and peripheral blood mononuclear cells (PBMCs) were obtained from healthy donors. Plasma and PBMCs were obtained by apheresis under the approved UHN REB protocol 05-0956-C. Potential donors gave written consent and underwent eligibility assessment including transmissible disease bloodwork, a health assessment questionnaire and physical assessment. Eligible donors underwent 5–15 L aphereses and plasma and PBMCs were collected. Plasma was processed by centrifugation to eliminate residual cells and frozen; leukopaks were processed by Ficoll density centrifugation to enrich for PBMCs and slow-speed centrifugation to eliminate platelets before cryopreservation. Serum and plasma were also obtained from Hemochromatosis donors undergoing therapeutic phlebotomy (UHN REB 06-0129-CE). Donors gave written consent and their blood was also tested for transmissible diseases. Serum was processed by clot formation and centrifugation; plasma was processed by the addition of 10 U/ml heparin (Leo Pharma) and centrifugation to eliminate residual cells. Commercially available human serum from Gemini Bio-Products was used for TIL cultures from initial specimens (specimens #M1 – M26), cryopreservation medium, and TILs undergoing the rapid expansion protocol.

### Histocompatibility leukocyte antigen (HLA) typing

HLA typing was performed by the Toronto General Hospital HLA lab by Polymerase chain reaction-reverse sequence specific oligonucleotide (PCR-RSSO).

### Media

For complete medium (CM), the following were added to Iscove's modified Dulbecco's medium (IMDM) (Lonza): 10% human serum or plasma (see below for additional details), 25 mM HEPES, 100 U/ml penicillin, 100 µg/ml streptomycin (Lonza), 10 µg/ml gentamicin sulfate (Lonza), 2 mM L-glutamine (Lonza), 5.5×10^−5^ M 2-mercaptoethanol, 6000 IU/ml human recombinant IL-2 (Chiron). For CM with plasma, plasma from 5–7 healthy donors were pooled together, filtered (0.2microns), and frozen in aliquots. Plasma aliquots were thawed and used to make CM. Aliquots of CM were then frozen until use. Aliquots were thawed before use, and then leftover thawed CM was kept at 37°C for a maximum of three days for further use and then discarded. IL-2 ELISAs showed no decrease in the IL-2 concentration using this procedure (data not shown). Plasma or CM with plasma was always thawed rapidly at 37°C and care was taken to avoid the formation of cryoprecipitates at 4°C. For enzyme dissociation medium, the following were added to IMDM: 1 mg/ml collagenase, 100 ug/ml DNase I and 30 U/ml hyaluronidase (Sigma-Aldrich), 10 ug/ml gentamicin sulfate, 2 mM L-glutamine, 1.25 µg/ml amphotericin B, 100 U/ml penicillin and 100 µg/ml streptomycin (Lonza). Cryopreservation medium was composed of 10% Cryoserv (Bioniche)/90% human serum (Gemini Bio-Products).

### TIL culturing

Methods for TIL culturing were the same as those used by the Rosenberg group. Several methods were used to process tissue: 1) enzymatic dissociation, 2) fine needle aspirates, 3) mechanical dissociation using a Medimachine (Becton Dickinson) and/or 4) directly plating small tissue fragments. For enzymatic dissociation, tissue was first minced into ∼1 mm^3^ pieces and then incubated on a stir plate at room temperature until tissue was dissociated (1–18 hours). After passing through a 100micron nylon mesh, cells were washed extensively before plating. For fine needle aspirates, cells were collected through a 23 gauge needle and then washed. For mechanical dissociation, small tissue pieces were loaded into Medicons and then dissociated according to the manufacturer's instructions. Cells were then subjected to Ficoll gradient centrifugation and the leukocyte-enriched layer collected for TIL growth. Single cell suspensions were plated at 1×10^6^ total cells per well, in 24-well tissue culture plates. For TIL growth from tissue fragments, one 1 mm^3^ fragment was placed into each well of 24-well plates. Cells were cultured in 2 ml per well of CM (containing 6000 IU/ml of human recombinant IL-2) in a 37°C, 5% CO_2_, humidified incubator. After the first week in culture, 1 ml medium from each well was replaced with fresh CM three times a week. Wells were maintained at a cell concentration of 0.5–2×10^6^ cells/ml. Each independent TIL culture was generally derived from 1–2 parental wells and upon subsequent expansions, all daughter wells were combined, mixed and re-plated.

### Flow cytometry

Cells were stained at 4°C for 30 minutes in buffer (2% fetal calf serum/0.05% sodium azide/PBS) containing antibodies, washed, and resuspended in 1% paraformaldehyde/PBS. Antibodies used included: CD3-phycoerythrin (PE), CD4-fluorescein isothiocyanate (FITC), CD8-peridinin chlorophyll protein (PerCP), CD56-allophycocyanin (APC), CD19-FITC, CD14-PerCP-Cyanine5.5. Data was acquired on a FACSCalibur flow cytometer (BD) and analyzed using FlowJo software.

### Immunohistochemistry (IHC)

4micron formalin-fixed paraffin-embedded sections were dewaxed in 5 changes of xylene and brought down to water through graded alcohols. Slides were pretreated with pepsin (1% pepsin in 0.01 N HCl (pH 2.0), 15 mins at 37°C). Endogenous peroxidase and biotin activities were blocked respectively using 3% hydrogen peroxide and avidin/biotin blocking kit (Vector labs.). After blocking for 15 mins with 10% normal goat serum, sections were incubated accordingly at room temperature with anti-human CD3 antibody (Dako) at 1∶100 for 1 hour. This was followed by 30 mins each with a biotinylated linking reagent (ID labs.) and horseradish peroxidase-conjugated ultrastreptavidin labeling reagent (ID labs.). After washing well in PBS, colour development was done with freshly prepared NovaRed solution (Vector labs.). Finally, sections were counterstained lightly with Mayer's hematoxylin, dehydrated in alcohols, cleared in xylene and mounted in Permount.

### Functional assays

TIL cultures were assayed for reactivity against the following panel of target cells, depending on availability and HLA-type: 1) autologous tumor cells (cryopreserved single cell suspensions from original tissue specimen and/or melanoma tumor cells propagated in culture (in CM without IL-2) from the original tissue specimen), 2) melanoma cell lines (kind gifts from M. Dudley and S. Rosenberg): 526 mel (HLA-A*0301/0201), 624 mel (HLA-A*0301/0201), 888 mel (HLA-A*0101/2402), 938 mel (HLA-A*0101/2402) (all expressing Melan-A/MART-1 and gp100), 3) T2 lymphoblastoid cells (HLA-A*0201+, TAP-deficient) pulsed for 2 hours with 1 µM Melan-A/MART-1-derived peptide (aa27–35), 1 µM gp100-derived peptide (aa209–217), or unpulsed. Melan-A/MART-1-specific and gp100-specific T cell lines were used as positive control T cells. Stimulation of TILs with 10 ng/ml Phorbol myristate acid (PMA) and 500 ng/ml ionomycin (Sigma) was used as a positive control for non-specific cytokine production. For IFN-γ production, 5×10^4^ TILs were co-cultured with 5×10^4^ target cells overnight at 37°C and supernatants were assayed for IFN-γ concentration by ELISA according to the manufacturer's instructions (Endogen). For cytotoxic T lymphocyte (CTL) assays, 2×10^3^ ^51^chromium-loaded target cells were plated in each well, together with TILs at various effector:target ratios. After 4–5 hours at 37°C, supernatants were transferred to LumaPlates (Perkin Elmer) and counted using a TopCount counter (Perkin Elmer). Maximal release was obtained using 1% Triton-X 100. Percent specific lysis was calculated as ((experimental release – spontaneous release)/(maximal release – spontaneous release))*100. Specific tumor reactivity was defined for IFN-γ as ≥200 pg/ml IFN-γ in response to autologous or HLA-A-matched target cells and >2-fold over background response of unstimulated TILs or against HLA-A-unmatched target cells. Specific tumor reactivity was defined for cytotoxicity as ≥20% specific lysis at a 60∶1 effector:target ratio against autologous or HLA-A-matched target cells and ≤10% specific lysis at a 60∶1 effector:target ratio against HLA-A-unmatched target cells.

### Rapid expansion protocol (REP)

TILs were thawed and rested in CM (containing 6000 IU/ml IL-2) for 1-3 days prior to initiating the REP. For initiation of REPs, the following were combined in T175 tissue culture flasks: 1×10^6^ TILs, 2×10^8^ allogeneic feeder cells (peripheral blood mononuclear cells pooled from 2–3 healthy donors and irradiated (50 Gy) before use), 30 ng/ml OKT3 (Janssen-Ortho), 3000 IU/ml IL-2 (Chiron) (no difference was seen in the expansion rate in the REP using 3000 IU/ml versus 6000 IU/ml of IL-2 (data not shown)), 75 ml CM and 75 ml AIM V serum-free medium (Gibco). REPs were maintained in a 37°C, 5% CO_2_, humidified incubator. On day 5, 80–90% of the media was replaced with fresh medium (50%CM/50%AIM V) and 3000 IU/ml IL-2. Cells were maintained at approximately 1×10^6^ cells/ml using AIM V with 5% human serum and 3000 IU/ml IL-2 on day 7 and plain AIM V medium and 3000 IU/ml IL-2 from day 8 onwards. Once cultures exceeded the T715 flask capacity, cells were transferred into 3 L Lifecell tissue culture bags (Baxter). This method was scaled down as necessary (e.g. 2×10^5^ TIL per T25 flask).
